# Measuring Equity in Access to Pharmaceutical Services Using Concentration Curve; Model Development

**Published:** 2015

**Authors:** Majid Davari, Elahe Khorasani, Zahra Bakhshizade, Marzie Jafarian Jazi, Mohsen Ghaffari Darab, Mohammad Reza Maracy

**Affiliations:** a *Department of Pharmacoeconomics and Pharmaceutical Administration, Faculty of Pharmacy, Tehran University of Medical Sciences, Tehran, Iran. *; b *Students’ Scientific Research Center, Tehran University of Medical Sciences, Tehran, Iran.*; c *Department of Health Services Management, School of Management and Medical Information, Isfahan University of Medical Sciences, Isfahan, Iran. *; d *Department of Pharmaceutics, School of Pharmacy and Pharmaceutical Sciences, Isfahan University of Medical Sciences, Isfahan, Iran. *; e *Department of Business Management, Mobarakeh Branch, Islamic Azad University, Mobarakeh, Isfahan, Iran.*; f *Department of Epidemiology and Biostatistics, School of Health, Isfahan University of Medical Sciences, Isfahan, Iran.*

**Keywords:** Equity in access, Pharmaceutical services, Concentration curve, Socioeconomic status, Poverty line, National minimum wage

## Abstract

This paper has two objectives. First, it establishes a model for scoring the access to pharmaceutical services. Second, it develops a model for measuring socioeconomic indicators independent of the time and place of study. These two measures are used for measuring equity in access to pharmaceutical services using concentration curve. We prepared an open-ended questionnaire and distributed it to academic experts to get their ideas to form access indicators and assign score to each indicator based on the pharmaceutical system. An extensive literature review was undertaken for the selection of indicators in order to determine the socioeconomic status (SES) of individuals. Experts’ opinions were also considered for scoring these indicators. These indicators were weighted by the Stepwise Adoption of Weights and were used to develop a model for measuring SES independent of the time and place of study. Nine factors were introduced for assessing the access to pharmaceutical services, based on pharmaceutical systems in middle-income countries. Five indicators were selected for determining the SES of individuals. A model for income classification based on poverty line was established. Likewise, a model for scoring home status based on national minimum wage was introduced. In summary, five important findings emerged from this study. These findings may assist researchers in measuring equity in access to pharmaceutical services and also could help them to apply a model for determining SES independent of the time and place of study. These also could provide a good opportunity for researchers to compare the results of various studies in a reasonable way; particularly in middle-income countries.

## Introduction

Equity is one of the fundamental principles of the healthcare systems worldwide (1). It is stated by the World Health Organization (WHO) (2) that equity in healthcare happens whenever health care resources are allocated according to patients' needs, and also payment for health services is made according to their ability to pay, regardless of the existing social attributes. Equity in the delivery of health services also means that all people have access to a minimum standard of health services if and when required irrespective of their economic, social, and demographic circumstances (3). But the key and important difficulties are: how such services should be financed, who should have access to which services and at what cost (4-5). Focusing on equity could help to answer these questions sensibly.

Equitable access to medicines is one of the essential challenges in developing and transitional countries (6-8). It is stated that about one-third of the populations around the world do not have normal access to essential medicines (9). Cameron showed that around 90% of the population in developing countries purchases their required medicines through out-of-pocket payments (10). This has made medicines the second largest family expenditure after food; and has made the cost of the medicines unaffordable for a huge number of people (10-11). Recent studies showed that out of pocket spending by the Iranian population for pharmaceutical services is now around 60% (8, 12-15). These studies revealed that equity in pharmaceutical services is going to be one of the main concerns in developing countries. This is mainly because the pharmaceutical management in these countries is faced with many challenges (16) including the amount of patients’ out of pocket expenses and the accessibility to the essential medicines. Nevertheless there is no strong published literature on equity in pharmaceutical services, particularly in middle income countries. 

The aim of this paper was to develop the methodology of equity measurement in access to pharmaceutical services by using concentration curve. We also focused on providing a new model for measuring socioeconomic indicators using city poverty line (CPL) and national minimum wage (NMW).

## Experimental

*Methods*

**Figure 1:**
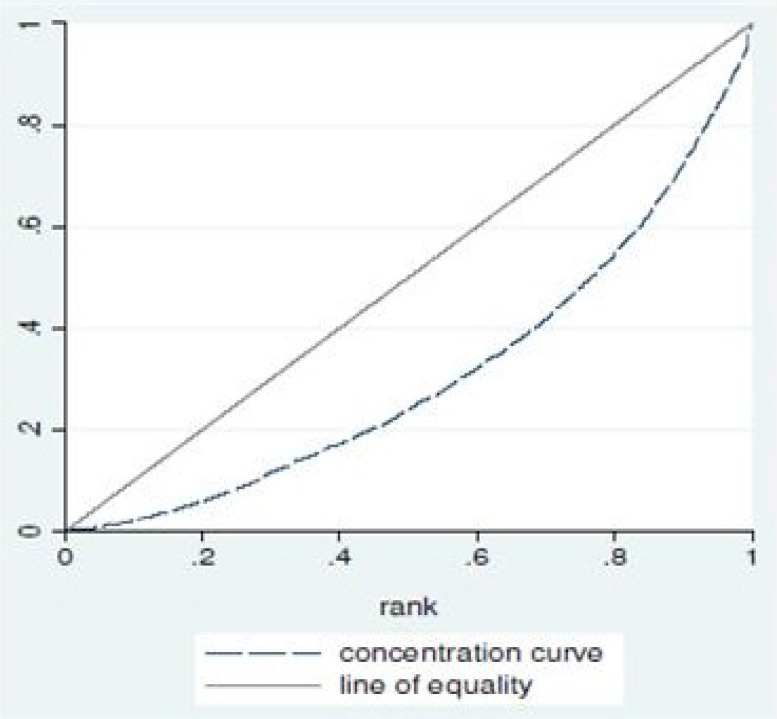
The classic diagram of concentration curve.

This methodological study was undertaken to develop a measurement of equity in access to pharmaceutical services. Measuring this equity requires comparing the indicators of access among different socioeconomic groups. Figure 1 shows the classic diagram of concentration curve. The Y-axis of this diagram shows the cumulative frequency percentage of access to pharmaceutical services; against a cumulative percentage of the population ranked by socioeconomic indicators, from extremely poor to the wealthiest socioeconomic status (SES), on the X-axis.

The equity line will be a 45-degree line, running from the bottom left-hand corner to the top right-hand corner (17-18). This straight line represents that the distribution of drugs is quite equitable and the curve below shows the concentration curve (19). 

The concentration index is equal to one minus twice the area under the concentration curve. This index is bounded between -1 and +1 and its sign is contractual; when concentration curve coincides with a 45^'^ line, access to pharmaceutical services is completely fair and concentration index will be zero. But, when all of the access goes to wealthiest people, this index will be +1 and if all the access goes to the poorest part of the society the index will be -1 (20).

It is clear that two criteria, including SES and the level of access to pharmaceutical services related to each class, are used in measuring the concentration index (19). The following formula is applied to measure this index (21):

C = (P_1_L_2_ –P_2_L_1_) + (P_2_L_3_ –P_3_L_2_) + ....... + (P_T-1_L_T_ –P_T_L_T-1_) 

Where P is the cumulative percentage of the population ranked by socio-economic status, T is the level of socio-economic class, and L is cumulative frequency percentage of access in each socio-economic class.

Thus we need to measure the level of access to pharmaceutical services in one hand, and determine the socioeconomic status of the subjects in the other hand. Concentration curve and its index, which are perhaps the most widely used measures to gauge inequity in healthcare (22-25) is considered for determining the level of equity in pharmaceutical services.

*Access to pharmaceutical services*

A comprehensive search was conducted on Pubmed, Embase and Google Scholar to find out any appropriate indicator/s for measuring access to pharmaceutical services, particularly in middle income countries. But, we did not find any relevant and suitable indicator for this purpose. Therefore we first prepared an open-ended questionnaire and distributed it to 30 academic experts to get their ideas for forming of the access elements and for assigning the scores to each element based on the Iranian pharmaceutical system. Secondly a concept-focused group were undertaken to finalize the numbers and the scores of each elements in the questionnaire (in this context is a checklist). Third, as the questions were objective, there was no need to check the reliability of the questionnaire (checklist), but its validity was verified by the academic experts. These academics were from various disciplines including pharmaceutical, heath economics, epidemiology, and health care management. This could help to measure the cumulative frequency percentage of access to pharmaceutical services through the concentration curve. The samples were selected purposely to cover various opinions. 

*Socioeconomic Status (SES)*

Better understanding about how and why SES relates to equity and access to health care in measurement of SES, received increasing attention in recent years (26-32). Generally SES is defined as the deferential access to desired resources (32). It is a multidimensional theoretical construct that covers a variety of social and financial circumstances (33-34). SES is measured by indicators including education, occupation, income, wealth and place of residence (35-36).

Selection of indicators which are used for the individuals' classification in a society is one of the major problems in socioeconomic studies (37); and integrating different indicators in determining SES is usually recognized as complex and multidimensional (38-39).

Each indicator represents a particular aspect of social stratification, which might be relevant to dissimilar health outcomes reasonably (39-40). But it is important to note that most of the SES indicators are, to some extent, correlated with each other (31, 40).

Tailoring the choice of SES indicators to the objectives of the study is very important (31). The selected indicators for categorizing the groups should be relevant to the purposes of the study. They should reflect identifiable subgroups of the population that require particular attention because their underlying social characteristics give them less opportunity to be healthy than their more advantaged counterparts (41).

In order to identify proper indicator/s for determining the SES of the patients, a widespread search was conducted on Pubmed, Embase and Google Scholar. Consequently several indicators were selected and considered in this study based on the extensive literature (26, 28, 31, 38-39, 42-44) and concept-focused group. These indicators include income, occupation, education, home status and family size. 

We first determined the average weight of each indicators and their impact on measuring SES through asking the experts opinions. We then distributed the selected indicators to academics, which were familiar with the subject, and asked them to weight the indicators based on Stepwise Adoption of Weights (SAW) which is considered as a simple method in multi-objective decision making (43, 45). The procedure of using this method involves the following four steps:

1. Determining the weight of each indicator 

The weights used to construct the indicators were derived from focused group discussion. The socioeconomic indicators were given to experts for weighting the value of each criteria based on skirt scoring. Then the impact percentage of each factor on SES for each person was calculated. 

2. Determining the score of each indicator 

For ranking and scoring the groups which were associated with each indicator, focused group discussion were used. 

3. Calculation of detailed individual scores

The detailed scores of the individuals were obtained by multiplying the desired person's character in the indicator weight. 

4. Calculation of individual final score 

The final individual score was derived from the sum of detailed individual scores, which could determine the SES of each person clearly. 

## Results

The systematic review of the published literature showed that there were not many published studies about equity in access to pharmaceutical services. The initial choice of the interviewees consisted of 45 academic experts, mainly from pharmaceutical discipline. Following the first contacts, 35 individuals agreed to complete and return our self-administered questionnaire, but only 30 questionnaires were actually completed and returned to us (85.7%). 

The collected results of this study are presented in two main subtitles; measuring access to pharmaceutical services and determining socioeconomic status. 

*Measuring access to pharmaceutical services*

Pharmaceutical system in each country has its own characteristics which may make it different from that of other countries. According to the experts' opinions 9 indicators were selected for assessing the access to pharmaceutical services based on the Iranian pharmaceutical system, which is impartially similar to many other pharmaceutical systems in middle-income countries. The indicators were scored by the subjects based on discomfort and annoyance caused by unavailability of the prescribed medicine. The finalized indicators and their scores are showed in Table 1. The higher the scores are the more comfortable is the access to the pharmaceutical services and vice versa. With the help of these scores the percentage of cumulative access to pharmaceutical services is evidently calculable. We supposed that the prescribed medicines are recommended based on the patients’ needs. 

Putting all indicators and their scores together, the calculation of the cumulative frequency percentage of access to pharmaceutical services and drawing Y- axis of the concentration curve diagram (Figure 1) become very straight forward. 

**Table 1:** Pharmaceutical access indicators

**No.**	**Access status**	**score**
1	All prescribed medicines are available in any pharmacy	100
2	Patient is forced to change his/her drug, but the alternative drug is available in any pharmacy	90
3	Patient has to go to a specific public pharmacy to obtain his/her medicine/s	80
4	The prescribed medicines are obtained with several visits to pharmacies	70
5	Patient has to trip to a bigger city to obtain his/her medicine/s	60
6	Patient has to trip to a bigger city and to a specific public pharmacy to obtain his/her medicine/s	50
7	Patient must trip to the capital city to take his/her medicine/s	30
8	Patient has to take his/her medicine from black market	10
9	The prescribed medicine is not available at all	1

*Measuring SES*

The results of ranking and scoring indicators for measuring SES are presented in Tables 2-9. 

**Table 2:** The average weight and the percentage of impact on SES.

**No.**	**Indicator**	**Weight**	**Impact on SES (%)**
1	Income	4.5	30
2	Occupation	4.0	27
3	Education	3	20
4	Home status	2.0	13
5	Family size	1.5	10

**Table 3:** Income classification based on city poverty line (C.P.L.).

**Groups**	**Monthly household income**	**Score**
extremely poor	Income ≤ 1/2 C.P.L.*	1
	1/2C.P.L.< Income ≤C.P.L.	2
Poor	C.P.L.< Income ≤2C.P.L.	3
Moderate	2C.P.L.<Income ≤3C.P.L.	4
	3C.P.L.< Income ≤ 4 C.P.L.	5
	4C.P.L.< income ≤ 5 C.P.L.	6
Wealthy	C.P.L.< Income	7

* City Poverty Line

**Table 4:** The ranks and scores of people occupation

**Scores**	**Ranks**	**Occupation categories**
Large and moderate landowners, Top-level managers, Professionals	High	6
Lower managers, Semiprofessionals, Vendors, Artisans	Average	3
low-wage industrial workers, officers, and retail sellers, Services workers, Unemployed, Pensioners	Low	1

**Table 5:** Education classification

**Level of education**	**Score**
Under High School Diploma	1
High School Diploma- Bachelor of Science	4
Master of Sciences and above	7

**Table 6:** The rank of homeowners based on price and surface size

**Home Surface (HS)**	**Score**	**Price per m** ^2^	**Score**	**Homeowner group** ^1^	**Score**
HS ≤ 100 m ^2^	1	Lowest price	1	Homeowner 1	1-5
100 m ^2^<HS ≤ 200 m ^2^	2	Low price	3	Homeowner 2	6-15
200 m ^2^<HS ≤ 300 m ^2^	3	Medium price	5	Homeowner 3	16-29
300 m ^2^<HS ≤ 400 m ^2^	4	High price	7	Homeowner 4	30-39
HS > 400 m ^2^	5	Highest price	9	Homeowner 5	40-45

1: Homeowner group= Home surface (HS) score* Price per m^2^ score

**Table 7:** The rank of tenants based on national minimum wage (NMW).

**Tenant group**	**Rent**
Tenant 1	Rent < 1/3 NMW
Tenant 2	1/3 NMW ≤ Rent < NMW
Tenant 3	NMW ≤ Rent <2NMW
Tenant 4	2 NMW ≤ Rent ≤5 NMW
Tenant 5	Rent >5 NMW

**Table 8:** The final scores of the home status

**Home status**	**Final scores**
Tenant 1	1
Tenant 2	2
Tenant 3	3
Homeowner1	4
Homeowner 2	5
Tenant 4	6
Homeowner 3	7
Tenant 5	8
Homeowner 4	9
Homeowner 5	10

**Table 9:** The scores of family size

**Family size**	**Scores**
FS ≤ 2	8
FS = 3	5
FS = 4	3
FS ≥ 5	1

*The weight of each indicator*

The selected indicators from the literature review [26, 28, 31, 38-39, 42-44], which were income, occupation, education, home status and family size, were given to the experts for weighting based on skirt scoring. The average weight and the percentage of the impact of each factor on SES were extracted from the questionnaires. The results are presented in Table 2 in descending order of importance. 

*Income*

Income received the highest weight for measuring SES from the subjects of our study (Table 2). Income is also considered as one of the most important factors in determining social stratification which represents the most direct measure of individual circumstances (46). In health studies, income is usually interpreted as one of the key factors influencing individual health through a direct impact on available financial resources needed for health care utilization. The association between income and individual health can be of a reverse causality type, which means that poor health experience a loss of income (40).

As total income reflects what households can actually spend on their day to day cares, this was used for social stratification of the population. The level of absolute poverty line of the city that the research was conducted in was used to categorize monthly household income. The poverty line defines the level of consumption (or income) needed for a household to escape poverty and the requirement to fulfill basic needs (47-48). Considering the experts’ opinions and economic context of Iran, which is considered as a middle-income country, 4 groups and 7 categories of income were selected to determine the SES of the individuals based on the city poverty line. The results are presented in table 3.

*Occupation*

Occupation is strongly related to income and is widely used for social stratification. It also has a direct relationship with material resources and health and may be related to health outcomes in a straight line (40). 

To facilitate occupation classification, different occupational schemas were reviewed (49-51). The most common feature of all schemas was that the majority of them had created a tripartite classification for occupations. We then integrated experts' opinions in order to reach a degree of consensus on the given scores to different occupation groups. Table 4 shows the ranking and scoring of individual occupation. 

*Education*

Education is one of the important indicators in determining individuals’ circumstances. Many studies showed that the level of individual education could influence their job opportunities and consequently their income. It could also affect their health behaviors and their lifestyle. The experts classified the educational level of individuals to three and scored them from 1 to 7 (Table 5). 

*Home status*

Home status is discovered by asking participants if they or their spouse owned a house or apartment or whether they rented one from the market. In order to score the home status of the individuals, it is easier and more practical to rate and compare the situation of homeowners and tenants in a single model. Thus we divided the evaluation process into 4 stages to establish a single model for determining the home status of individuals. 

In the first stage individuals were separated into two groups; homeowners and tenants. 

In the second stage the homeowners were ranked based on the size and price of their homes. This stage was conducted in three steps. First, we classified the home surface into five groups; equal or less than 100^m2^ to larger than 400^m2^ and scored them from 1 to 5. Second, we also classified the price per square meter of the homes into 5 groups, lowest to highest quintile of local price per square meter. Then the experts were asked to score each quintile from 1 (lowest price) to 9 (highest price). In the third step the score of homeowners were determined by multiplying their scores in the two previous steps. Afterward the homeowners were scored from 1 to 45 and were ranked into quintile groups from 1 to 5. The results of stage 2 are presented in Table 6. 

The status of the tenants was decided in the third stage. The NMW was considered as references for classifying the tenants’ status. On the basis of the market price and the experts’ opinions the tenants were classified into 5 groups. The results of this classification are presented in Table 7. 

In the fourth stage, taking into account the experts' opinions, the situation of homeowners and tenants was ranked, scored and compared in a single model. Table 8 showed the final result of stage 4 in home status measurement. 

*Family size*

For income to be comparable across households, family size (FS) or the number of people dependent on the reported income should be collected (40, 52-53). 

FS is attained from a summary of questions asking participants to give the number of spouses, parents, siblings, children or children-in-law, grandchildren, or other relatives living in their household. The subgroups and their scores, which are resulted from the experts' views, are presented in Table 9. 

*Calculation of individual scores*

Final individual scores are derived from the sum of the detailed individual scores. Taking into account all SES indicators and their scores, the lowest possible score is 100 (when all parameters are set at their minimum scores, one), and the highest possible score is 720 (when all parameters are at their highest value). These scores are classified into 5 groups, from the lowest to the highest quintile of the final scores, for determining the SES of individuals. These include extremely poor (scores between 100 and 224), poor (scores between 225 and 348), moderate (scores between 349 and 472), good (scores between 473 and 596) and wealthy (scores between 597 and 720) classes. 

For example, if the score of a subject in income is 6, in occupation 3, in education 7, in home status 5, and in family size 5, his/her final score would be 516 and he/she would be placed in "good" SES. 

## Conclusion

The purpose of this methodological paper is to improve the methodology of equity measurement in access to pharmaceutical services using concentration curve. We developed a set of indicators for measuring access to pharmaceutical services and also introduced a new method of measuring socioeconomic indicators using CPL and NMW. 

The five important findings which emerged from this study are as follows:

1) The weight and the score of 9 indicators for assessing the access to pharmaceutical services are established.

2) The average weight and the percentage of the impact of each socioeconomic indicator on the socioeconomic status of individuals are suggested. 

3) A model based on CPL is introduced for income classification. 

4) A model based on NMW is developed for rent ranking. 

5) A single model is accomplished for comparing homeowners and tenants.

These findings may help researchers in developing more practical methods for measuring SES and pharmaceutical equity, independent of the time and place of study. This improvement will provide a good opportunity for researchers to compare the results of various studies in a reasonable way; particularly in middle-income countries. 
